# HsCRP in Patients with Acute Exacerbation of Chronic Obstructive Pulmonary Disease

**Published:** 2011-10-01

**Authors:** S A Alavi, F Soati, K Forghanparast, H Amani

**Affiliations:** 1 Respiratory and TB research Center, Guilan University of Medical Sciences, Rasht, Guilan, Iran

**Keywords:** C-reactive protein, Diagnosis, Chronic obstructive pulmonary disease

## Abstract

**Background:**

Chronic obstructive pulmonary disease (COPD) is currently the fourth leading cause of death in the United States. As there is systemic as well as local inflammation in COPD patients and evaluating the stage of the disease is not possible by spirometery alone, we evaluated High-Sensitivity C-reactive Protein (HS-CRP) in a group of COPD patients as an available and cost effective auxiliary marker in determining COPD stages.

**Methods:**

In a cross-sectional study in 160 COPD patients who were admitted for exacerbations in Razi Hospital in Rasht, Data on patients' demographic characteristics, pulmonary function test (PFT) and laboratory results consist of arterial blood gases and HSCRP levels were analyzed.

**Results:**

A significant positive correlation was seen between serum HSCRP level and stages of the disease (as GOLD criteria). There was a significant relationship between HSCRP level and patients' sex, BMI, and smoking history in a way that men and smokers showed higher and patients with normal BMI showed lower HSCRP levels. The patients with higher PCO2 also showed a higher level of serum HSCRP.

**Conclusions:**

This survey supports the role of HSCRP as a simple auxiliary marker in staging and determining the prognosis of COPD for early management.

## Introduction

Chronic obstructive pulmonary disease (COPD) is defined as a disease state characterized by airway limitation that is not fully reversible, is usually progressive and is associated with an abnormal inflammatory response of the lungs to inhaled noxious particles or gases.[1][2][3]In COPD, subsets of patients may have dominant features of chronic bronchitis, emphysema, or asthma. The result is irreversible airflow obstruction. COPD is a disorder that causes a huge degree of human suffering and currently is the fourth leading cause of death in United States. Development of the 20th century included the widespread use of spirometry, recognition of airflow obstruction as a key factor in determining disabilityin COPD.[4][5][6]

Patients with COPD experience a systemic inflammation which can be assessed by measuring inflammatory mediators like C-reactive protein (CRP).[7] Recently, High-Sensitivity C-reactive Protein (HSCRP) measuring methods have made it possible to assess this protein in lower levels of inflammation. Prognostic value of HS-CRP is proved in cardiovascular diseases.[8] The cellular composition of the airway inflammation in COPD is predominately mediated by neutrophils. Macrophages also play an important role through macrophage-derived matrix metalloproteinase (MMPs). Mounting evidence supports that the dysregulation of apoptosis and defective clearance of apoptotic cells by macrophages play predominant role in airway inflammation.[9][10]

The aim of the present study was to assess HSCRP as a cost-effective auxiliary marker other than spirometery in determining severity of COPD and better control of disease prognosis in patients with exacerbations, and also explore the co-variants such as age, gender, co-morbidities, smoking, PO2 and PCO2 in these patients.

## Materials and Methods

This was a descriptive cross-sectional study which was performed on COPD patients who were referred to Respiratory Department of Razi Hospital, Guilan, North Province of Iran because of COPD acute exacerbation during 2008-2009. COPD patients whose diseases were diagnosed by specialists and confirmed by spirometery (as gold criteria) entered the survey. They were excluded if their diseases were not confirmed by FEV1/FVC<70% or FEV1/VCmax<70% in Pulmonary Function Test (PFT), or if they had a history of asthma, connective tissue disorders (e.g. Rheumatoid Arthritis, Systemic Lupus Erythematosus, …), inflammatory diseases (e.g. Inflammatory Bowel Disease) or a known malignancy.

Data were collected on patients’ demographic characteristics, co-morbidities (hypertension, congestive heart failure, diabetes mellitus, Hyperlipidemia) by history, physical exam and echocardiography, smoking habits and number of exacerbations. The following laboratory tests were performed for all patients in the same lab: HS-CRP with the same kit at the beginning of admission; Arterial blood gases (e.g. PO2, PCO2); Pulmonary function tests (e.g. FEV1, FVC, FEV1/FVC), severity of disease that was determined by Gold Criteria and blood samples (for Hb, HCT).

* HS-CRP was measured quantitavely by microplateimmunoenzymometric assay and interpreted in the ranges of <3 mg/l (normal and low risk) and =3 mg/l (high risk). Data were analyzed using descriptive statistics (e.g percentage and mean) and comparing means (e.g. Chi Square, T and Pearson's correlation tests). P<0.05 was considered significant.

## Results

In this study, 160 known COPD patients (134 males and 26 females) with exacerbation were assessed. Baseline characteristics of patients were shown in Table 1. Their mean age (SD) of sample was 65.19±10.66 years. Among the participants, 127 patients (79.4%) were smokers, 92 patients had at least one co-morbidity. Regarding severity of disease according to gold criteria, one patient was in stage I, 34 (21.3%) in stage II and most of them (125 patients) were in stage III and IV. Mean serum HS-CRP level was 11.65±15.03 mg/l. Among the samples, 54 (33.8%) patients showed mild and 52 (32.5%) revealed moderate hypoxemia (PO2 60-80 mmHg and 40-60 mmHg respectively) and most of them (61.2%) showed PCO2=45mmHg. The most common comorbidities were heart failure and hypertension (77.5% of all co-morbidities).

Also 31.3% of males and 34.6% of females showed anemia (HCT<39% in males and <36% in females).

Men and smokers showed significantly higher levels of HS-CRP (p=0.014 and p= 0.043, respectively). Of the factors in Table 1, patients’ BMI , PaCO2, and stage of disease (as gold criteria) showed significant association with HS-CRP level (p=0.008, p=0.011, and p=0.0001 respectively); but there was no relationship between age groups, amount of smoking (pack/year), any of the co-morbidities and number of exacerbations during last year and HS-CRP level.

**Fig. 1:**
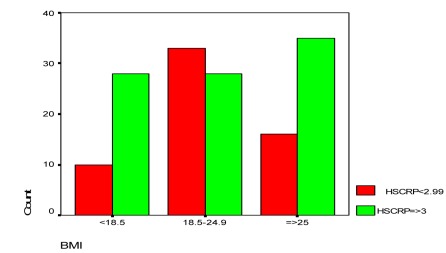
BMI and HS-CRP relationship in COPD patients

The relationship between BMI and HS-CRP level showed that patients with normal BMI (18.5-25 kg/m2) mostly had lower levels of serum HS-CRP while underweight and overweight patients tended to have high risk levels of HS-CRP (Figure 1). Of the factors in Table 2, PO2, mean of Hb and HCT did not show any correlation with HS-CRP level, but by increasing PCO2 in Arterial Blood Gas, patients showed higher HS-CRP too. Table 2 shows Pearson’s correlations between variables and HS-CRP.

**Table 1:** Baseline characteristics of the COPD patients and their relationship with HS-CRP^a^.

**Variables^a^**	**Mean±(SD)**	***P* value^b^**
Age, year	65.19 (10.66)	0.318
BMI	22.75 (4.97)	0.008
Pack/year history in smokers	48.11 (24.01)	0.189
Number of exacerbations	1.02 (1.18)	0.581
PaO2 (mmHg)	65.64 (21.30)	0.772
PaCO2 (mmHg)	52.21 (15.59)	0.011
FEV1(% predicted)	36.83 (14.30)	0.0001
Hb (g/dl)	13.01 (2.09)	0.53

^a^ Chi-square test was used to determine the relationships

^b^ P value <0.05 considered as significant

**Table 2:** Correlations between different variables and HS-CRP in COPD patients.

**Variables**	**Pearson's correlation(r)**	***P* value**^a^
PaO2 (mmHg)	0.023	0.772
PaCO2 (mmHg)	+0.201	0.011
Hb (g/dl)	0.050	0.53
FEV1(% predicted)	-0.392	0.0001

^a^ P value <0.05 was considered significant

## Discussion

The main finding of our study was that serum HSCRP level was significantly correlated to stage of disease (as gold criteria). Also we found a significant relationship between patients' serum HS-CRP level and their gender, BMI, smoking habits and PaCO2 in exacerbation state. In the present study, more males showed high risk levels of HS-CRP than females, similar to Breyer's study (2005-2007)[11] which showed an increased likelihood for highly elevated CRP in males.Overall, considering this fact that in the present study COPD gold stages were significantly higher in males, maybe the reason of higher CRP levels in men was higher stages of disease in these patients.

Although it seems that COPD patients experience some degrees of metabolic changes and muscle wasting, and this fact is associated with higher levels of inflammatory markers and hypermetabolic state, in this study and some similar investigations overweight and obese patients (measured by BMI) showed higher levels of CRP (as an inflammatory marker) than normal weight patients. In a study by Attaran et al. (2008) in Mashhad, there was no relationship between BMI and HS-CRP level.[12] But in Juan de Torres et al. study (2008) in Spain, patients with higher CRP levels showed a higher BMI.[13] Also in Breyer et al.'s study,[11] obese patients showed higher CRP levels than normal weight patients. They also analyzed Fat Free Mass Index in these patients and did not find any relationship between CRP and Fat Free Mass Index. So they did not get to a definite conclusion. In de torress et al.'s study (2002-2004), BMI was correlated directly with CRP and they concluded that inflammatory markers such as TNF-a and CRP, had different behavior, relating to malnutrition or perhaps reflecting depletion of different components (FFMI versus fat mass, respectively).[14]

The novel finding of the present study was that underweight patients showed higher HS-CRP levels than normal weight patients (that shows muscle wasting effects of systemic inflammation) as well as obese and overweight patients. Although in 3 BMI groups, majority of patients showed high risk of CRP levels, patients with normal BMI as Chi-square analysis were most likely to have low risk HS-CRP levels (p=0.008).

Increased CRP levels in obese COPD patients, which can also be seen in normal CAD patients may in part be explained by the fact that increased adipose tissue induced increased levels of adipocytokynes which in turn may stimulate production of CRP in the liver. Indeed, IL-6 and TNF-a were shown to stimulate the production of CRP through facilitating hepatocytes.[11] More studies are needed to help resolve the controversial findings.

In the present study, more percentage of smokers showed high risk levels of HS-CRP (p=0.043), but there was no significant difference in Pack/Year history of patients between different HS-CRP levels (p=0.189). de Torres et al. in two different studies did not show any significant relationship between amount of smoking and HS-CRP levels, although smokers showed higher levels of HS-CRP.[13][14] Halvani et al. (2006) also did not find any significant relationship in HS-CRP level between smokers and non-smokers. In their opinion, although smoking had a role in initiation of inflammatory process in COPD patients, it was not the leading cause of increased inflammatory markers.[15]

It is now widely accepted that smoking is the main risk factor for reduced pulmonary function in COPD. Compounds of tobacco smoke also penetrated into the blood stream, and were associated with a higher prevalence of coronary artery disease, endothelial dysfunction, and high serum concentrations of markers of systemic inflammation. High CRP concentrations may reflect the effects of smoking, but this observation that changes in CRP concentrations were associated with FEV1 decline in those who had never smoked can imply this fact that this association may not be due to smoking.[16] It should be noticed that not all cases develop inflammatory reaction following smoking and only some of them would show this reaction which can be due to genetic differences.[17] In the present study, the effect of other environmental factors which can act similar to smoking was not analyzed.

In the current study we did not find significantly higher HS-CRP levels in those who had one or more co morbidities (Coronary Artery Diseases, Congestive Heart Failure, Hypertension, and Diabetes Mellitus) or had one or more exacerbation histories during the last year. In the literature, there are strong arguments for CRP increasing thrombotic risk and cardiovascular deaths.[9] But the findings of our study did not support this fact. Donaldson et al.[18] in their study showed no higher prevalence of ischemic heart disease in participants who had a COPD outcome than those free of an event. Also in their study, none of the inflammatory markers were associated with a greater prevalence of heart diseases. Torres et al. (2004) did not find any significant relationship between CRP levels and cardiovascular risk factors and diseases too.[14] Maybe such as some other researches, we can say that CRP is a strong predictor of COPD outcomes, independent of cardiovascular diseases.

We should take it into account that we did not matched patients by drugs which they used as statins and we also did not actively assess cardiovascular diseases and risk factors except Congestive Heart Failure which was confirmed by echocardiography. Others were diagnosed by history and physical exam and patients were not followed for future outcomes.

Another strange findings of the present study just like Halvani et al. and Pinto-Plata et al.'s studies [15][17] was not a relationship between number of exacerbations during last year and HS-CRP level. We did not have any justification for this finding. Maybe it is due to some limitations as low sample size, just studying exacerbations during a single year and not considering different cultural states of patients.

Among laboratory findings we found significant relationship between HS-CRP and PaCO2 p<0.0001) but not about PaO2 and Hb. Attaran et al.,[12] similarly found a positive correlation between HS-CRP and PaCO2 and no relationship was noticed between HS-CRP and PaO2 in patients with COPD due to toxicity with sulfur mustard. Breyer et al. did not find any significant difference in PaO2 between different levels of CRP.[11] The key point comes from Bircan et al.’s study which found negative correlation between PaO2 and HS-CRP in control group and patient with stable COPD but not in patients with exacerbation.[19] As we see in our study and Bircan et al.’s study, PaO2 was not a valuable measurement while exacerbations and perhaps Pa- CO2 was a better tool for making decisions. Maybe the reason is that these patients mostly are in respiratory distress and immediately inhale considerable amount of O2 in the beginning of admission and maybe this is just a bias due to different factors which can interfere in final result of an ABG test.

The main finding of the current study was the correlation between FEV1% (stage of disease) and HS-CRP level in COPD patients with exacerbation. In Halvani et al.’s study, serum CRP level was significantly higher in COPD patients.[15] de Torres et al. (2004) showed a correlation between CRP and FEV1 in stable COPD patients.[14] Shaaban et al. in a crosssectional analysis (2001) found a significant relationship between FEV1% and CRP, although longitudinal analysis didn’t support it.[16] Rezayitalab et al. similarly found a negative correlation (r= 0.341) between CRP and HSCRP.[20] Also Bircan et al. got to a negative correlation between these two factors (r=0.417).[19] We found a negative correlation between HS-CRP and FEV1% in exacerbation too (r=0.392).

The reasons for the inverse association between systemic inflammation and reduced pulmonary function are unclear but several mechanisms maybe involved.First, reduced lung function may be responsible for systemic inflammation. Like hepatocytes, inflammatory lung or pulmonary epithelial cells, have been shown to express CRP and IL-6. An alternative mechanism–reverse causation- cannot be excluded. High levels of cytokines and acute phase reactants in peripheral circulation maybe a cause rather than a consequence of poor lung function.[16]

Considering CRP as a systemic inflammatory marker and systemic inflammation as an important factor in determining outcomes of COPD patients, and increase in higher stages, it seems that this marker can be useful as an auxiliary marker other than Pulmonary Function Test in follow up of patients and assessing their status and effects of treatments.

Limitations of the present study were low sample size, not analyzing drugs including statins and corticosteroids, and not having a matched control group. Totally, the current study supports a relationship between HS-CRP as an inflammatory marker and stage of COPD (as gold criteria) and PaCO2 and also implies that increasing inflammation in higher stages is not due to cardiovascular diseases and is due to the disease itself. This study confirms that HS-CRP increases following smoking but there is a question whether smoking is the main cause of increase in CRP level in the patients or not and this study supports the role of obesity in increase of inflammation in COPD too.
